# Stabilization and Steam Activation of Petroleum-Based Pitch-Derived Activated Carbons for Siloxane and H_2_S Gas Removal

**DOI:** 10.3390/ma18245563

**Published:** 2025-12-11

**Authors:** Geon-Hee Lee, Jin Kyun Kang, Byong Chol Bai, Yong-Wan Park

**Affiliations:** 1 Korea Institute of Convergence Textile (KICTEX), Iksan 54588, Republic of Korea; 2 Division of Energy Engineering, Daejin University, Pocheon 11159, Republic of Korea

**Keywords:** petroleum-based pitch, activated carbon, oxidative stabilization, mesopore, siloxane and H_2_S removal

## Abstract

Activated carbons were synthesized from petroleum-based pitch and evaluated for the removal of trace siloxanes and hydrogen sulfide (H_2_S) from gas streams. Oxidative stabilization followed by steam activation produced high specific surface area with enlarged mesoporosity (BET up to 1620.9 m^2^ g^−1^), as confirmed by N_2_ sorption (BET/PSD), SEM, and elemental analysis. A GC/MS-based fixed-bed assay using 5 g of adsorbent, a 100 mL min^−1^ challenge flow, and a 30 min readout was employed to quantify performance under consistent conditions. Under these tests, siloxanes were not detected at 30 min, and H_2_S decreased to 0.38 ppm. Samples with greater mesopore volume while retaining high surface area showed higher 30 min removal. Surface-chemistry analysis indicated that oxygen functionalities introduced during stabilization facilitated pore development during subsequent steam activation without substantial loss of area. Taken together, the textural and adsorption results present a coherent picture in which a micro/mesopore architecture supports siloxane and H_2_S control under the stated test conditions. The study records the key testing parameters and performance values to enable practical comparison of petroleum-pitch-derived activated carbons for gas purification.

## 1. Introduction

Volatile siloxanes and hydrogen sulfide (H_2_S) are critical trace contaminants in gas-purification trains for biogas upgrading, landfill-gas utilization, and industrial off-gases [1,2,3]. Siloxanes oxidize to silica-like deposits that abrade engines and deactivate downstream catalysts, while H_2_S promotes corrosion and catalyst poisoning at low parts-per-million levels [4,5,6]. Because both concentrations and damage thresholds are low, sorbents must provide reliable uptake together with adequate mass transfer to accessible pore space and sufficient chemical tolerance [7].

A broad set of approaches has been investigated to mitigate these species, including catalytic oxidation, liquid absorbents, metal-oxide chemisorbents and composites, membrane separations, and physical adsorbents used as pre-polishing or polishing steps [8,9,10]. Within this landscape, activated carbons remain attractive owing to high specific surface area, tunable pore structures, thermal and chemical stability, and established manufacturing routes [11,12,13].

For activated carbons, pore architecture is a principal determinant of performance [14]. Micropores (<2 nm, IUPAC) offer strong adsorption potentials at low partial pressures, whereas mesopores (2–50 nm) enhance diffusion and accessibility for larger or condensable molecules such as cyclic siloxanes and help sustain removal in mixed streams [15,16,17]. Achieving a balanced micro/mesopore network and describing its influence on short-time removal under defined test conditions is therefore central to material selection [18].

During stabilization and subsequent steam activation, oxygen-containing functional groups (such as carbonyl, carboxyl, and phenolic species) serve as preferential gasification sites because they possess lower bond dissociation energies than the surrounding carbon matrix. These oxygenated sites are selectively etched during activation, allowing micropores to widen and evolve into mesopores, thereby enabling more effective diffusion of bulky siloxane molecules.

For many conventional activated carbons, precise control of mesoporosity is challenging because precursor compositions vary, activation outcomes are highly condition-dependent, and gains in surface area often come at the expense of yield or mechanical integrity [19,20]. Pitch-based activated carbons present a practical alternative [21]. Studies in electrochemical double-layer capacitors and related energy-storage fields have developed strategies for tuning mesoporosity and pore connectivity, and petroleum-based pitch, by virtue of its high fixed-carbon content and relatively narrow heteroatom distribution, offers advantages for structural control without sacrificial templating [22,23].

However, translating pitch-derived mesopore design to harmful-gas adsorption, specifically for siloxanes and H_2_S, has received little focused attention [24,25]. Gas-cleaning applications impose distinct constraints compared with energy-storage contexts, including short-time performance under defined flow and residence conditions, possible competitive adsorption in multicomponent streams, humidity effects, and the need to limit pressure drop while maintaining capacity; these factors motivate materials-focused studies that relate measurable textural and surface-chemical descriptors to standardized adsorption readouts so that samples can be compared on an equivalent basis [26,27].

In this study, oxidative stabilization is applied to guide pore development during activation with the aim of obtaining pitch-derived activated carbons suited to siloxane and H_2_S control. We characterize pore and surface properties by nitrogen sorption (BET surface area and pore-size distribution), scanning electron microscopy, elemental analysis, and X-ray photoelectron spectroscopy, and we quantify short-time removal using a fixed-bed GC/MS assay operated under consistent conditions. The findings of this study provide valuable insights into the potential application of pitch-based activated carbons with tailored mesopore characteristics for the removal of volatile siloxanes and H_2_S in gas-purification settings.

## 2. Materials and Methods

### 2.1. Materials

In this study, petroleum-based pitch (petroleum based pitch, Smart Korea, Daejeon, Republic of Korea) with a softening point of 215 °C was used. The petroleum-based pitch exhibited a hydrogen-to-carbon (H/C) atomic ratio of 0.650 and an aromaticity of 0.285. For the gas adsorption tests, hydrogen sulfide (H_2_S, 1000 ppm; Rigas, Daejeon, Republic of Korea) and siloxane gas (D5 siloxane gas, 10 ppm; Rigas, Daejeon, Republic of Korea) were introduced separately through independent adsorption lines, with N_2_ used as the balance gas in each case.

### 2.2. Activated Carbon Manufacturing with Pitch

The petroleum-based pitch precursor with a softening point of 215 °C was stabilized to introduce oxygen functional groups prior to carbonization. Approximately 10 g of the pitch sample was uniformly spread on an alumina plate and placed in a tubular furnace with a controlled air flow for stabilization. The temperature was increased at a rate of 2 °C/min to the target stabilization temperatures of 180, 200, 220, 240, 260, 280, and 300 °C, and maintained for 1 h under an air atmosphere. Among these, the sample stabilized at 260 °C was selected for subsequent carbonization. Carbonization was performed in a tubular furnace under a continuous N_2_ flow. The temperature was raised to 700 °C at a rate of 10 °C/min and held for 1 h to obtain carbonized pitch. Subsequently, steam activation was carried out to develop the porous structure. The carbonized sample was heated at a rate of 10 °C/min up to 900 °C under N_2_ atmosphere, followed by steam injection at a rate of 1 and 2 cm^3^/min per 10 g of each sample. The activation was maintained for 90 min and 120 min, respectively, to control the degree of activation. After activation, the obtained samples were cooled to room temperature and dried in an oven at 80 °C for 24 h.

### 2.3. Siloxane and H_2_S Gas Adsorption

**Figure 1:**
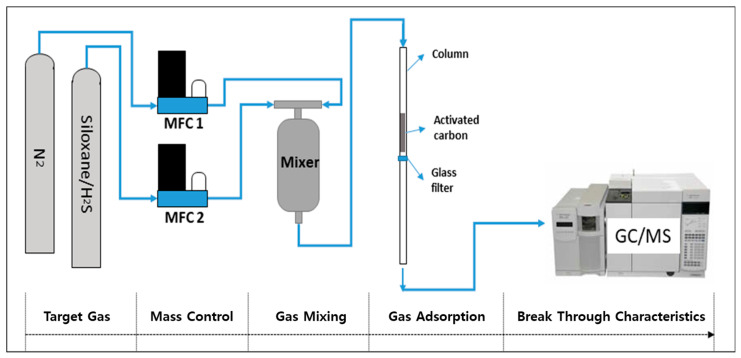
Schematic diagram of the experimental setup.

To quantitatively evaluate the gas adsorption performance of the activated carbon prepared via the physical activation process, calibration curves were first established for the target gases, D5 siloxane (10 ppm) and H_2_S (1000 ppm). Approximately 5 g of the activated carbon sample was packed into a glass column, and the mixed gas was introduced at a flow rate of 100 cm^3^/min. The gas concentration after 30 min of adsorption was analyzed using a gas chromatograph-mass spectrometer (GC/MS, Agilent 7890A coupled with 5975C MSD, Agilent Technologies, Inc., Santa Clara, CA, USA). For siloxane gas, the calibration curve was obtained by mixing siloxane gas with N_2_ to prepare standard concentrations of 1.25, 2.5, 5, and 10 ppm, and the corresponding GC/MS response values were recorded. For H_2_S gas, the calibration curve was generated by mixing H_2_S gas with N_2_ at standard concentrations of 100, 500, and 1000 ppm, and the GC/MS responses were measured accordingly. A schematic diagram of the experimental setup is shown in Figure 1.

### 2.4. Characterization of ACs

To examine the variation in oxygen content after the stabilization process, elemental analysis (EA) was performed. The surface morphology of the samples before and after stabilization, as well as after activation, was observed using scanning electron microscopy (SEM). In addition, X-ray diffraction (XRD) analysis was conducted to investigate the structural evolution with respect to activation time. To determine the pore structures of the samples, we utilized Brunauer–Emmett–Teller (BET) specific surface area analysis (Micromeritics Instrument Co., ASAP2020, Norcross, GA, USA). Prior to the measurements, all samples were degassed at 300 °C for 6 h under vacuum. Overall, the comprehensive analysis of adsorption data obtained across a relative pressure (P/P_0_) range spanning from 10^−5^ to 1 and conducted at 77 K provides valuable insights into the evolving pore structures of samples under different activation conditions. Because the samples were micropore-dominant, pore-size distributions were obtained using the NLDFT model. The pore-size distribution was calculated using the NLDFT carbon slit-pore kernel in the range of 0.35–30 nm. The lower bound corresponds to the kinetic diameter of nitrogen (0.364 nm), below which pores cannot be resolved by N_2_ adsorption at 77 K. Micropore volumes were determined from NLDFT analysis and the mesopore volumes were calculated as the difference between total pore volume and micropore volume. To quantitatively assess the degree of infusibility of the pitch after the infusibilization process, a softening point system (DP90, Mettler Toledo, Greifensee, Switzerland) was used to measure the infusibility at various stabilization temperatures. Each sample was analyzed twice, and the average values were reported. Finally, the gas adsorption properties of the activated carbon were analyzed by monitoring the gas concentration for 30 min using GC/MS.

## 3. Results and Discussion

### 3.1. Effect of Stabilization Temperature on the Softening and Infusibility Behavior of Pitch

The infusibility of the SP 215 pitch was evaluated by examining its softening point and weight change as a function of stabilization temperature. As shown in Table 1, the sample stabilized at 180 °C exhibited an average softening point of 322.7 °C, whereas no measurable softening point (N.D.) was detected for the samples treated at 200 °C or higher. This result indicates that partial oxidative crosslinking occurs at 180 °C, while stabilization at ≥200 °C leads to complete infusibilization of the pitch, preventing any softening upon subsequent heating. This behavior is associated with the progressive formation of oxygen-containing functional groups during the stabilization process. The incorporation of oxygen into the pitch matrix promotes oxidative crosslinking reactions, thereby increasing the degree of infusibility. For SP 215 pitch, such oxygen functionalization initiates below the softening point of the untreated pitch, allowing sufficient crosslinking to occur prior to melting and consequently enabling efficient stabilization [28]. The weight variation before and after stabilization is summarized in Table 2. As the stabilization temperature increased from 180 °C to 300 °C, the sample mass increased from 0.261 g to 0.610 g, corresponding to substantial oxygen uptake. This consistent weight gain confirms the temperature-dependent nature of oxidative stabilization, leading to enhanced thermal stability and improved infusibility of the SP 215 pitch.

### 3.2. Oxygen Incorporation Behavior of SP 215 Pitch During Stabilization

EA was conducted to examine the change in elemental composition of the SP 215 pitch as a function of stabilization temperature, and the results are summarized in Table 3. As the stabilization temperature increased, the oxygen content of the pitch progressively rose from 0.87 wt.% in the pristine SP 215 pitch to 13.99 wt.% at 300 °C. This continuous increase in oxygen-containing species indicates the progression of oxidative reactions during the infusibilization process. In particular, the sample stabilized at 300 °C exhibited the highest oxygen content, suggesting that oxidation proceeds more extensively at higher temperatures and leads to a more densely crosslinked and oxygen-rich structure [29]. Such oxygen incorporation is closely related to the development of infusibility and enhanced thermal stability in the stabilized pitch.

During stabilization, oxygen incorporation into the pitch occurs through a series of well-known oxidative reactions that convert the thermoplastic pitch into an infusible, crosslinked structure. As the pitch is exposed to oxygen at elevated temperatures, surface and near-surface radicals are generated through dehydrogenation, which facilitates the formation of oxygen-containing functional groups such as carbonyl, carboxyl, and ether linkages. These oxygen functionalities subsequently promote intermolecular crosslinking by bridging adjacent aromatic units, thereby increasing molecular rigidity. In addition, oxygen-induced crosslinking suppresses melting behavior by forming thermally stable C–O–C and C=O bonds. This progressive increase in oxygen uptake, as confirmed by elemental analysis, reflects the extent of oxidative crosslinking and corresponds to the transition of the pitch from a softening, fusible material to a stabilized, infusible precursor suitable for subsequent carbonization.

During the subsequent steam activation stage, these oxygen-containing functional groups are expected to play a critical role in promoting pore development [30]. At high activation temperatures, steam reacts with carbon through the following gasification pathways:

C + H_2_O → CO + H_2_ (water–gas reaction)

C + 2H_2_O → CO_2_ + 2H_2_ (steam gasification reaction)

CO + H_2_O → CO_2_ + H_2_ (water–gas shift reaction)

These reactions generate reactive gases such as CO, CO_2_, and H_2_, which actively gasify the carbon matrix. The oxygen functional groups introduced during stabilization act as reactive sites for steam–carbon interactions, facilitating gas evolution and accelerating the gasification of carbon. This promotes the opening of closed pores, the enlargement of existing pores, and the formation of a well-developed pore network within the material [31]. Therefore, the increased oxygen incorporation observed in the stabilized SP 215 pitch is anticipated to enhance the efficiency of steam activation, ultimately improving the porosity and textural properties of the resulting activated carbon.

### 3.3. Morphological Evolution and Pore Formation Behavior of SP 215 Pitch

**Figure 2:**
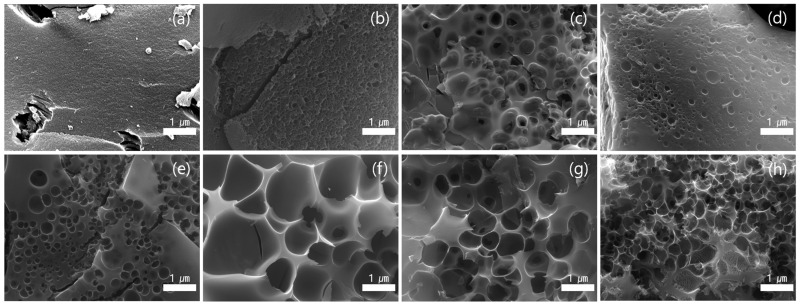
SEM images of SP 215 pitch stabilized at different temperatures: (**a**) pristine SP 215, (**b**) 180 °C, (**c**) 200 °C, (**d**) 220 °C, (**e**) 240 °C, (**f**) 260 °C, (**g**) 280 °C, and (**h**) 300 °C.

The surface morphologies of the SP 215 pitch samples stabilized at different temperatures were examined by SEM, as shown in Figure 2. At lower stabilization temperatures (Figure 2a,b), the surface remained relatively dense and smooth, with only minor cracks visible and no significant pore-related features. As the stabilization temperature increased (Figure 2c,d), numerous small bubble-like voids began to appear, indicating the onset of internal gas evolution and oxidative crosslinking. At even higher stabilization temperatures (Figure 2e–h), these voids developed into well-defined, spherical hollow domains with thin surrounding carbon walls. The abundance and enlargement of these bubble-like structures suggest progressive gas release and structural rearrangement within the pitch matrix. Such morphological evolution demonstrates the formation of pore precursors that can grow into more developed pore structures during the subsequent steam activation stage.

### 3.4. Surface Morphology of Activated Carbon Under Different Activation Times

**Figure 3:**
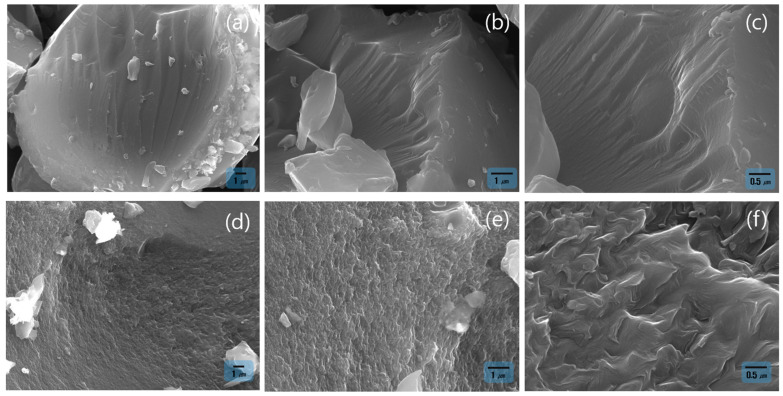
SEM images of activated carbon derived from SP 215 pitch after activation for (**a**–**c**) 90 min and (**d**–**f**) 120 min at magnifications of 5000×, 10,000×, and 20,000×, respectively.

The surface morphologies of the activated carbons derived from SP 215 pitch, which was stabilized at 260 °C, were examined by SEM image, as shown in Figure 3. After 90 min of steam activation (the steam flow rate fixed at 1 cm^3^/min, Figure 3a–c), the samples maintained a relatively smooth and compact surface, showing only shallow grooves and limited surface disruption, indicating that the activation reaction was still at an early stage. In contrast, the samples activated for 120 min (Figure 3d–f) exhibited markedly roughened and textured surfaces accompanied by extensive carbon etching, layer peeling, and the emergence of irregular cavity-like features. These morphological changes demonstrate that increasing the activation time leads to more advanced structural evolution driven by steam–carbon reactions, resulting in a more developed and complex surface architecture.

### 3.5. XRD Analysis of Activated Carbon with Different Activation Times

**Figure 4:**
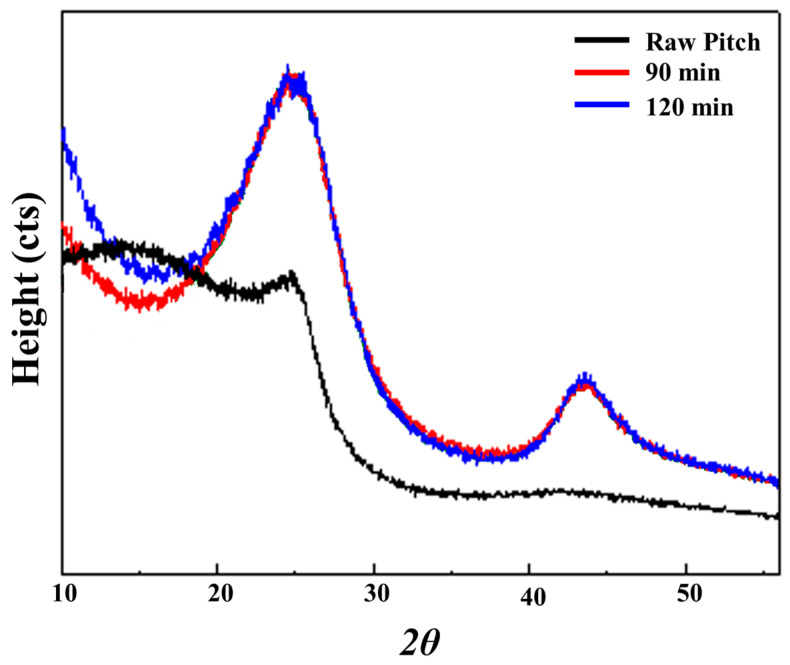
XRD patterns of activated carbon derived from SP 215 pitch after activation for 90 and 120 min.

XRD analysis was performed to examine the structural evolution of the activated carbons prepared at different activation times (90 min and 120 min). As shown in Figure 4, both samples exhibited two broad diffraction peaks near 2θ ≈ 24–25° and 43°, corresponding to the (002) and (100) planes of turbostratic carbon [32]. The broad nature of these peaks indicates that the activated carbon largely consists of amorphous carbon with limited graphitic stacking. According to Table 4, the (002) peak for the 90 min sample was located at 24.75°, whereas that for the 120 min sample shifted to 24.60°. This decrease in 2θ resulted in an increase in the interlayer spacing (d002) from 3.593 Å to 3.616 Å, suggesting that the carbon layers became partially expanded due to enhanced gasification and the removal of loosely bound species during prolonged activation. In addition, the peak intensity decreased from 3128 to 2840 counts, and the FWHM increased from 6.90 to 7.18, indicating reduced stacking order and the generation of a more disordered carbon structure. These structural changes imply that longer activation durations promote increased disorder and defect formation, which are favorable for pore development [33]. The increased interlayer spacing and disorder observed after 120 min of activation correlate well with the SEM results showing a more textured and wrinkled surface morphology.

During activation, the (002) and (100) reflections gradually broaden and decrease in intensity, indicating increased structural disorder in the carbon matrix. Such peak broadening is typically associated with a reduction in crystallite coherence lengths, even in the absence of explicit Scherrer quantification. This progressive disordering is consistent with the development of micropores and the widening of existing pores as activation time increases.

### 3.6. Textural Properties of Activated Carbon Under Different Activation Conditions

**Figure 5:**
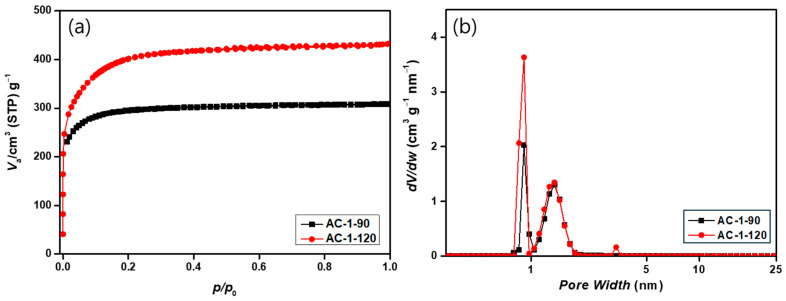
N_2_ adsorption–desorption isotherms and pore size distributions of activated carbon prepared at a steam flow rate of 1 cm^3^/min for different activation times: (**a**) isotherms and (**b**) pore size distributions.

**Figure 6:**
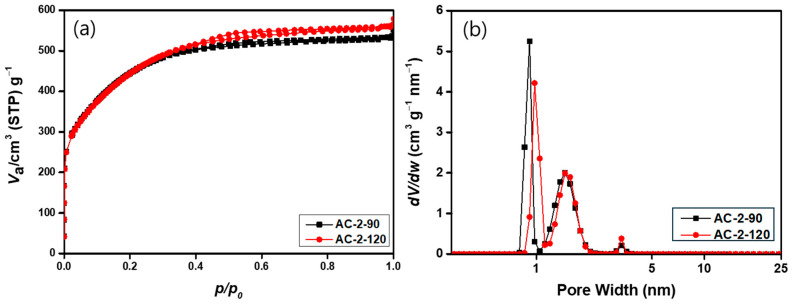
N_2_ adsorption–desorption isotherms and pore size distributions of activated carbon prepared at a steam flow rate of 2 cm^3^/min for different activation times: (**a**) isotherms and (**b**) pore size distributions.

The specific surface area and pore characteristics of the activated carbons were analyzed by N_2_ adsorption–desorption isotherms and pore size distribution, as shown in Figure 5 and Figure 6 and Table 5 and Table 6. Through this analysis, we aim to investigate the effects of steam flow rate and activation time. When the steam flow rate was fixed at 1 cm^3^/min and the activation time increased from 90 to 120 min, the BET surface area rose markedly from 1145.9 to 1402.1 m^2^/g, accompanied by an increase in the total pore volume from 0.4775 to 0.6141 cm^3^/g. Both micropore and mesopore volumes increased simultaneously, indicating that prolonged activation promoted deeper steam–carbon gasification and the development of a more complex pore network. The N_2_ isotherms exhibited typical Type I characteristics [34], confirming that the activated carbons were primarily microporous. However, the pore size distribution revealed a meaningful increase in mesopore formation, with mesoporosity rising from 16.6% to 18.9% as activation time increased. This trend suggests that continuous steam etching not only generates new micropores but also widens existing narrow pore channels, leading to the conversion of some micropores into mesopores [35]. The average pore size also increased slightly from 8.8 Å to 9.2 Å, supporting the occurrence of structural expansion within the carbon matrix as activation progressed. These textural changes correlate well with the SEM observations (Figure 2), where the 120 min sample exhibited a more etched, wrinkled, and roughened surface morphology. Overall, the activation time strongly governs the balance between pore formation and pore widening. Therefore, prolonged activation leads to a hierarchical pore structure with improved accessibility, which is advantageous for adsorption performance and mass transport in practical applications [36].

At a fixed steam flow of 2 cm^3^/min, prolonging the activation time from 90 to 120 min slightly increased the BET surface area from 1617.7 to 1620.9 m^2^/g and the total pore volume from 0.8216 to 0.8638 cm^3^/g. Although the overall increase was modest, the mesopore volume showed a noticeable rise from 0.1798 to 0.2142 cm^3^/g, indicating continued mesopore development during prolonged activation. The isotherms maintained a typical type I pattern, and the pore-size distribution showed a sharp peak below 2 nm with only a minor contribution from larger pores, confirming that micropores remained dominant. Overall, the results suggest that extending the activation time enhances mesoporosity while maintaining high micropore volume, resulting in a well-balanced micro–mesoporous structure suitable for adsorption and electrochemical applications.

### 3.7. Adsorption Characteristics of Siloxane and H_2_S Gases on Activated Carbon

(1) Siloxane gas adsorption behavior

**Figure 7:**
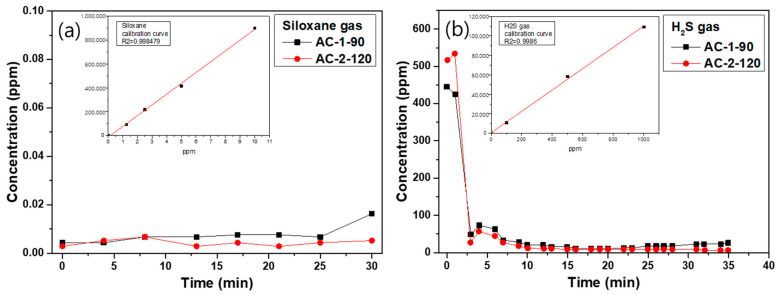
Adsorption behavior of (**a**) siloxane gas and (**b**) H_2_S gas analyzed by GC/MS.

For the siloxane adsorption experiment, standard gas mixtures were prepared by diluting siloxane with nitrogen to concentrations of 1.25, 2.5, 5, and 10 ppm. The GC/MS response values at these concentrations were used to construct a calibration curve, which exhibited excellent linearity (R^2^ = 0.998). A 10 ppm siloxane stream was then passed through the packed column at 100 cm^3^/min, and the outlet concentration was monitored for 30 min. As shown in Figure 7a, AC-1-90 maintained 0 ppm throughout most of the 30 min test, but a slight increase to 0.02 ppm was detected at the final time point, indicating an extremely delayed breakthrough. In contrast, AC-2-120 maintained 0 ppm for the entire 30 min, demonstrating complete removal without measurable breakthrough. This difference correlates strongly with the structural characteristics of carbons. AC-2-120, which possesses a higher BET surface area and significantly greater mesopore volume (0.2142 cm^3^/g vs. 0.1798 cm^3^/g in AC-2-90), provides more accessible adsorption sites and improved diffusion pathways. These structural advantages facilitate deeper penetration of siloxane molecules into the pore network, thereby suppressing early breakthrough. This indicates that prolonged activation enhances the hierarchical pore structure, ultimately improving siloxane adsorption performance [37].

(2) H_2_S gas adsorption behavior

For H_2_S, calibration curves were prepared at 100, 500, and 1000 ppm by dilution with nitrogen, demonstrating high linearity (R^2^ = 0.999). When 1000 ppm H_2_S was fed into the column, both samples exhibited an immediate drop in concentration due to rapid initial uptake. However, their breakthrough characteristics differed substantially. AC-1-90 began to break through after approximately 20 min, and the outlet concentration progressively increased, reaching 23 ppm at 35 min. This relatively early saturation reflects its lower pore volume and insufficient mesopore development, which restricts gas transport and limits the accessible adsorption capacity. On the other hand, AC-2-120 maintained exceptionally low outlet concentrations, ending at only 0.38 ppm at 35 min without showing breakthrough. The enhanced performance is attributed to the larger mesopore fraction (41.0%) and increased total pore volume (0.8638 cm^3^/g), which together enable faster intra-particle diffusion and greater utilization of internal adsorption sites. These features foster more efficient mass transfer and significantly improve H_2_S removal efficiency compared to AC-1-90 [38]. Collectively, the H_2_S results confirm that prolonged steam activation not only increases the total pore volume but also optimizes micro-mesopore balance, which is critical for achieving stable and long-duration adsorption under high contaminant loads.

## 4. Conclusions

In this study, activated carbon with well-developed mesoporous features was successfully prepared from SP 215 pitch through stabilization, carbonization, and steam activation. Increasing the stabilization temperature promoted oxygen incorporation from 0.87 wt.% to 13.99 wt.%, facilitating oxidative crosslinking and enhancing the infusibility of the pitch. SEM and XRD analyses confirmed that higher stabilization temperatures and longer activation times increased structural disorder and expanded the interlayer spacing, contributing to progressive pore formation and enlargement. The steam activation conditions significantly affected the development of the pore structure. Increasing the steam flow rate from 1 to 2 cm^3^/min raised the BET surface area from 1145.9 to 1617.7 m^2^/g, while extending the activation time increased the total pore volume from 0.4775 to 0.6141 cm^3^/g at 1 cm^3^/min and from 0.8216 to 0.8638 cm^3^/g at 2 cm^3^/min. The mesopore fraction also increased from 16.6% to 24.8%, indicating that steam activation effectively promotes both micropore formation and mesopore widening. These structural improvements enhanced diffusion pathways and facilitated better access to internal adsorption sites. The adsorption behavior of siloxane and H_2_S clearly reflected the influence of pore structure. AC-2-120, which possessed a larger mesopore volume and pore dimensions more compatible with the molecular sizes of the target gases, maintained complete removal of siloxane for 30 min and achieved an H_2_S outlet concentration of only 0.38 ppm at 35 min. In contrast, AC-1-90 exhibited delayed siloxane breakthrough and earlier H_2_S saturation due to diffusion resistance associated with limited mesopore development. Overall, the performance of SP 215 pitch-derived activated carbon is determined by the balance between micropores and mesopores formed during stabilization and steam activation. The development of mesopores plays a particularly important role in reducing diffusion limitations and improving adsorption efficiency. These findings highlight the strong potential of SP 215-based activated carbon for biogas purification, especially for the removal of trace siloxanes and high concentrations of H_2_S.

## Figures and Tables

**Table 1:** Softening point variation of SP 215 pitch under different stabilization temperatures.

	NO.1 (°C)	NO.2 (°C)	Average (°C)
215-180	317.7	327.6	322.7
215-200	N.D.	N.D.	-
215-220	N.D.	N.D.	-
215-240	N.D.	N.D.	-
215-260	N.D.	N.D.	-
215-280	N.D.	N.D.	-
215-300	N.D.	N.D.	-

**Table 2:** Weight change of SP 215 pitch during stabilization at various temperatures.

	Weight Before Stabilization (g)	Weight After Stabilization (g)	Difference (g)
215-180	10.000	10.261	0.261
215-200	9.999	10.275	0.276
215-220	10.000	10.367	0.367
215-240	10.002	10.427	0.425
215-260	10.000	10.546	0.546
215-280	10.000	10.478	0.478
215-300	10.000	10.610	0.610

**Table 3:** Elemental composition of SP 215 pitch stabilized at various temperatures.

	SP 215	215-180	215-200	215-220	215-240	215-260	215-280	215-300
C (wt.%)	93.75	91.28	90.36	88.51	87.93	85.26	85.20	82.56
H (wt.%)	5.38	4.97	4.78	4.45	4.28	3.98	3.97	3.45
N (wt.%)	0	0	0	0	0	0	0	0
S (wt.%)	0	0	0	0	0	0	0	0
O (wt.%)	0.87	3.75	4.86	7.04	7.79	10.76	10.83	13.99

**Table 4:** XRD parameters of activated carbon derived from SP 215 pitch at different activation times.

	90 min	120 min
2*θ*	24.75	24.60
Height (cts)	3128.02	2840.29
FWHM	6.9	7.18
*d*-space (Å)	3.593	3.616

**Table 5:** BET surface properties of the activated carbons prepared at different activation times under a steam flow rate of 1 cm^3^/min.

Amount of Steam (cm^3^/min)	1	
Activation Time (min)	90	120
Sample Name	AC-1-90	AC-1-120
BET Surface Area (m^2^/g)	1145.9	1402.1
Total Pore Volume (cm^3^/g)	0.4775	0.6141
Micropore Volume (cm^3^/g)	0.3963	0.5036
Mesopore Volume (cm^3^/g)	0.0812	0.1105
Mesoporosity (%)	16.6	18.9
Average Pore Size (Å)	8.8	9.2

**Table 6:** BET surface properties of the activated carbons prepared at different activation times under a steam flow rate of 2 cm^3^/min.

Amount of Steam (cm^3^/min)	2	
Activation Time (min)	90	120
Sample Name	AC-2-90	AC-2-120
BET Surface Area (m^2^/g)	1617.7	1620.9
Total Pore Volume (cm^3^/g)	0.8216	0.8638
Micropore Volume (cm^3^/g)	0.6418	0.6496
Mesopore Volume (cm^3^/g)	0.1798	0.2142
Mesoporosity (%)	21.9	24.8
Average Pore Size (Å)	9.2	9.5

## Data Availability

The data presented in this study are available on request from the corresponding author due to the fact that the research project is still ongoing and has not yet been completed.

## References

[1] Moreno-Andrade I., Moreno G., Quijano G. (2020). Theoretical framework for the estimation of H_2_S concentration in biogas produced from complex sulfur-rich substrates. Environ. Sci. Pollut. Res..

[2] Konkol I., Cebula J., Świerczek L., Piechaczek-Wereszczyńska M., Cenian A. (2022). Biogas pollution and mineral deposits formed on the elements of landfill gas engines. Materials.

[3] Le Pera A., Sellaro M., Pellegrino C., Limonti C., Siciliano A. (2024). Combined pre-treatment technologies for cleaning biogas before its upgrading to biomethane: An Italian full-scale anaerobic digester case Study. Appl. Sci..

[4] Wang N., Tan L., Xie L., Wang Y., Ellis T. (2020). Investigation of volatile methyl siloxanes in biogas and the ambient environment in a landfill. J. Environ. Sci..

[5] Inaba M., Kuramoto K., Soneda Y. (2023). Effect of coexistence of H_2_S on production of hydrogen and solid carbon by methane decomposition using Fe catalyst. Int. J. Hydrogen Energy.

[6] Kim D., Kim K.H., Lim C., Lee Y.S. (2022). Carbon-coated SiOx anode materials via PVD and pyrolyzed fuel oil to achieve lithium-ion batteries with high cycling stability. Carbon Lett..

[7] Elwood M. (2021). The scientific basis for occupational exposure limits for hydrogen sulphide—A critical commentary. Int. J. Environ. Res. Public Health.

[8] Wu L., Zhu Y., Yuan J., Guo X., Zhang Q. (2024). Advances in adsorption, absorption, and catalytic materials for VOCs generated in typical industries. Energies.

[9] Pasichnyk M., Stanovsky P., Polezhaev P., Zach B., Šyc M., Bobák M., Jansen J.C., Přibyl M., Bara J.E., Friess K. (2023). Membrane technology for challenging separations: Removal of CO_2_, SO_2_ and NOx from flue and waste gases. Sep. Purif. Technol..

[10] Sharma R., Segato T., Delplancke M.P., Terryn H., Baron G.V., Denayer J.F., Cousin-Saint-Remi J. (2020). Hydrogen chloride removal from hydrogen gas by adsorption on hydrated ion-exchanged zeolites. Chem. Eng. J..

[11] Sun X., Zhao Y., Li Y., Guo Y., Xia L., Wang L., Xiang S. (2024). Promotional effect of Sr modification on the catalytic oxidation of hydrogen chloride to chlorine over Cu/Y zeolite catalyst. Chem. Phys..

[12] Wang C., Bai L., Zhao F., Bai L. (2022). Activated carbon fibers derived from natural cattail fibers for supercapacitors. Carbon Lett..

[13] Lim C., Kwak C.H., Jeong S.G., Kim D., Lee Y.S. (2023). Enhanced CO_2_ adsorption of activated carbon with simultaneous surface etching and functionalization by nitrogen plasma treatment. Carbon Lett..

[14] Li S., Tan X., Li H., Gao Y., Wang Q., Li G., Guo M. (2022). Investigation on pore structure regulation of activated carbon derived from sargassum and its application in supercapacitor. Sci. Rep..

[15] Zhang K., Sun J., Ma C., Luo S., Wu Z., Li W., Liu S. (2022). Effects of the pore structure of commercial activated carbon on the electrochemical performance of supercapacitors. J. Energy Storage.

[16] Cruz O.F., Campello-Gómez I., Casco M.E., Serafin J., Silvestre-Albero J., Martínez-Escandell M., Hotza D., Rambo C.R. (2023). Enhanced CO_2_ capture by cupuassu shell-derived activated carbon with high microporous volume. Carbon Lett..

[17] Gaj K. (2020). Adsorptive biogas purification from siloxanes—A critical review. Energies.

[18] Fundneider T., Alonso V.A., Abbt-Braun G., Wick A., Albrecht D., Lackner S. (2021). Empty bed contact time: The key for micropollutant removal in activated carbon filters. Water Res..

[19] Altwala A., Mokaya R. (2022). Modulating the porosity of activated carbons via pre-mixed precursors for simultaneously enhanced gravimetric and volumetric methane uptake. J. Mater. Chem. A.

[20] Moosavi S., Lai C.W., Gan S., Zamiri G., Akbarzadeh Pivehzhani O., Johan M.R. (2020). Application of efficient magnetic particles and activated carbon for dye removal from wastewater. ACS Omega.

[21] Kim M.I., Seo S.W., Kwak C.H., Cho J.H., Im J.S. (2021). The effect of oxidation on the physical activation of pitch: Crystal structure of carbonized pitch and textural properties of activated carbon after pitch oxidation. Mater. Chem. Phys..

[22] Shao H., Wu Y.C., Lin Z., Taberna P.L., Simon P. (2020). Nanoporous carbon for electrochemical capacitive energy storage. Chem. Soc. Rev..

[23] Ma Z.H., Yang T., Song Y., Chen W.S., Duan C.F., Song H.H., Tian X.J., Gong Z.Y., Liu Z.Y., Liu Z.J. (2024). A review of the catalytic preparation of mesophase pitch. New Carbon Mater..

[24] Negara D.N.K.P., Widiyarta I.M., Suriadi I.G.A.K., Dwijana I.G.K., Penindra I.M.D.B., Tenaya I.G.N.P., Sukadana I.G.K., Ferdinand A.S. (2023). Development of mesoporous activated carbons derived from brewed coffee waste for CO_2_ adsorption. EUREKA Phys. Eng..

[25] Tran V.T.L., Gélin P., Ferronato C., Mascunan P., Rac V., Chovelon J.-M., Postole G. (2019). Siloxane adsorption on activated carbons: Role of the surface chemistry on sorption properties in humid atmosphere and regenerability issues. Chem. Eng. J..

[26] Choleva E., Mitsopoulos A., Dimitropoulou G., Romanos G.E., Kouvelos E., Pilatos G., Beltsios K., Stefanidis S., Lappas A., Sfetsas T. (2023). Adsorption of hydrogen sulfide on activated carbon materials derived from the solid fibrous digestate. Materials.

[27] Vali S.A., Moral-Vico J., Font X., Sánchez A. (2024). Adsorptive removal of siloxanes from biogas: Recent advances in catalyst reusability and water content effect. Biomass Convers. Biorefinery.

[28] Yuan G., Li X., Xiong X., Dong Z., Westwood A., Li B., Ye C., Ma G., Cui Z., Cong Y. (2017). A comprehensive study on the oxidative stabilization of mesophase pitch-based tape-shaped thick fibers with oxygen. Carbon.

[29] Lee S.M., Lee S.H., Jung D.-H. (2021). Surface oxidation of petroleum pitch to improve mesopore ratio and specific surface area of activated carbon. Sci. Rep..

[30] Kim J.-H., Kim S.-H., Kim B.-J., Lee H.-M. (2023). Effects of oxygen-containing functional groups on the electrochemical performance of activated carbon for EDLCs. Nanomaterials.

[31] Chan H., Du Q., Yue C., Feng Y., Liu F., Li A. (2026). Hierarchical porosity engineering of biomass-activated carbon cathodes via dual-etching: Directional control of H_2_O_2_ electrogeneration and antibiotics degradation. Appl. Catal. B Environ. Energy.

[32] Dziejarski B., Serafin J., Hernández-Barreto D.F., Naumovska E., Sreńscek-Nazzal J., Klomkliang N., Tam E., Krzyżyńska R., Andersson K., Knutsson P. (2025). Tailoring highly surface and microporous activated carbons (ACs) from biomass via KOH, K_2_C_2_O_4_ and KOH/K_2_C_2_O_4_ activation for efficient CO_2_ capture and CO_2_/N_2_ selectivity: Characterization, experimental and molecular simulation insights. Chem. Eng. J..

[33] Zhou S., Tan L., Zhu T., Zhu H., Guo J., Li X., Dong Z., Zhang Q., Cong Y. (2025). Engineering optimal pore architecture and defect-rich structure in pitch-derived carbon for efficient sodium-ion storage. Chem. Eng. J..

[34] Wang J., Fu W., Wang L., Li Y., Li Y., Sui Z., Xu X. (2023). Modulation of pore structure in a microporous carbon for enhanced adsorption of perfluorinated electron specialty gases with efficient separation. Chem. Eng. J..

[35] Liu J., Zhang K., Wang H., Lin L., Zhang J., Li P., Zhang Q., Shi J., Cui H. (2022). Advances in micro-/mesopore regulation methods for plant-derived carbon materials. Polymers.

[36] Xiang Y., Lu L., Kottapalli A.G.P., Pei Y. (2022). Status and perspectives of hierarchical porous carbon materials in terms of high-performance lithium-sulfur batteries. Carbon Energy.

[37] Wen L., Liu J., Wang J., Wang M., Yang J., Liu Y., Zhang X. (2025). Insight into the adsorption behaviors of siloxane on activated carbon fibers. Microporous Mesoporous Mater..

[38] Chen L., Yuan J., Li T., Jiang X., Ma S., Cen W., Jiang W. (2021). A Regenerable N-rich hierarchical porous carbon synthesized from waste biomass for H_2_S removal at room temperature. Sci. Total Environ..

